# Biorefining of protein waste for production of sustainable fuels and chemicals

**DOI:** 10.1186/s13068-018-1234-5

**Published:** 2018-09-20

**Authors:** Si-Yu Li, I-Son Ng, Po Ting Chen, Chung-Jen Chiang, Yun-Peng Chao

**Affiliations:** 1 Department of Chemical Engineering, National Chung Hsing University, Taichung, 402 Taiwan; 2 Department of Chemical Engineering, National Cheng Kung University, Tainan, 70101 Taiwan; 3 Department of Biotechnology, Southern Taiwan University of Science and Technology, Tainan, 710 Taiwan; 4 Department of Medical Laboratory Science and Biotechnology, China Medical University, No. 91, Hsueh-Shih Road, Taichung, 40402 Taiwan; 5 Department of Chemical Engineering, Feng Chia University, 100 Wenhwa Road, Taichung, 40724 Taiwan; 6 Department of Health and Nutrition Biotechnology, Asia University, Taichung, 41354 Taiwan; 7 Department of Medical Research, China Medical University Hospital, Taichung, 40447 Taiwan

**Keywords:** Biorefinery, Protein waste, Biomass, Bio-based chemicals, Metabolic engineering

## Abstract

To mitigate the climate change caused by CO_2_ emission, the global incentive to the low-carbon alternatives as replacement of fossil fuel-derived products continuously expands the need for renewable feedstock. There will be accompanied by the generation of enormous protein waste as a result. The economical viability of the biorefinery platform can be realized once the surplus protein waste is recycled in a circular economy scenario. In this context, the present review focuses on the current development of biotechnology with the emphasis on biotransformation and metabolic engineering to refine protein-derived amino acids for production of fuels and chemicals. Its scope starts with the explosion of potential feedstock sources rich in protein waste. The availability of techniques is applied for purification and hydrolysis of various feedstock proteins to amino acids. Useful lessons are leaned from the microbial catabolism of amino acids and lay a foundation for the development of the protein-based biotechnology. At last, the future perspective of the biorefinery scheme based on protein waste is discussed associated with remarks on possible solutions to overcome the technical bottlenecks.

## Background

Our daily life currently dependent on fossil feedstock has been overshadowed by the global warming effect. This issue pressingly calls on the joined efforts of the international community towards climate change mitigation by reducing CO_2_ emission. One emergent and promising field arises and devotes to exploration of plant biomass-based fuels and chemicals for replacement of fossil resource-derived counterparts. Development of novel technology platforms for renewable production of energy and chemicals still remains a focus of research efforts [1–6]. However, the progress of the second-generation biofuel industry is fallen short of expectations because of technological bottlenecks and ecological issues associated with land use [7].

Replacement of the fossil fuels with biofuels used in the heavy transport sector is necessary to meet the CO_2_ reduction requirement committed at Paris Climate Conference in 2015 (COP21) [7]. A sustainable production of biofuels relies on lignocellulosic biomass. This renewable biomass can produce the waste residues exceeding 2 × 10^11^ tons per year worldwide [8]. Biomass stemming from dedicated energy crops and the waste streams of agro-food industries will be in a great demand because of the global incentives to the low-carbon alternatives. The expanding need for renewable feedstock is accompanied by the generation of a large volume of protein waste. As estimated, protein waste with 100 million tons per year could be generated if the use of biofuels (e.g., bioethanol and biodiesel) accounts for 10% of the global fuel demand [9]. The surplus protein waste necessitates recycling in a circular economy scenario. Valorization of biomass waste for various uses was estimated to rate bulk chemicals with the highest value of $1000 per ton of biomass, as compared to the value of transportation fuel ($200–400), cattle feed ($70–200), and electricity ($60–150) [8]. Therefore, it appears incentive to produce chemicals from protein waste [10].

This article provides a concise overview of the advances in refining protein waste for production of fuels and chemicals with special emphasis on the biotechnology platforms. The scope includes the available sources of feedstock, protein recovery and pretreatment, amino-acid catabolism, and the enzyme- and microbe-based production schemes of chemicals. As released from proteins, amino acids containing carbon skeletons with amino groups have functional similarities to the many petroleum-derived chemicals. The approach by enzymatic transformation (biotransformation) is straightforward and enables conversion of amino acids to bulk chemicals, particularly favorable for those with optical purity. On the other hand, microbes naturally utilize amino acids as food. The biomass-based schemes available for microbial production of chemicals can be transformed into those based on proteins. However, the development of both fields is still in infancy. This review is to provide an idea framework for future research efforts to this end.

## Potential sources of protein waste

### Protein source from crops

**Fig. 1:**
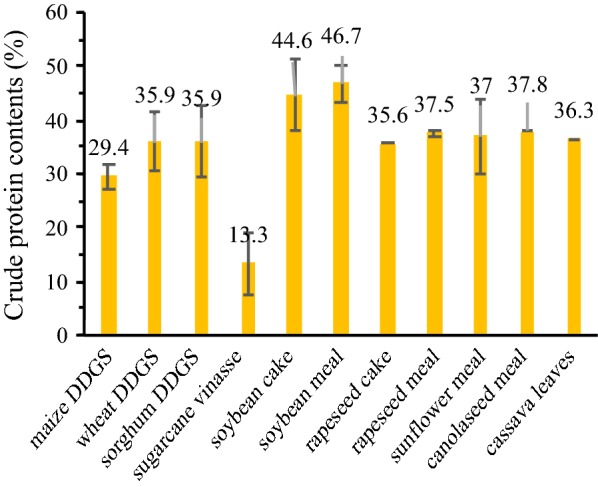
Crude protein content of biorefinery feedstock

The feedstock conventionally applied in biorefinery platforms mainly involves crops rich in sugars (e.g., sugarbeet and sugarcane), starch (e.g., cassava, maize, wheat, and sorghum), hemicellulose (e.g., switchgrass and coppice trees), and oil (e.g., *Jatropha* seed, palm, rapeseed, soybean, and sunflower seed). Waste streams resulting from the production of vegetable oil and biodiesel with oil crops comprise a very high protein content ranging between 40 and 60% (w/w) of the mass fraction, and their vital value is generally acknowledged as feedstuff. Nevertheless, *Jatropha* meal generated by the biodiesel production has a potential application for the chemical production, because it is inedible without detoxification. Recognized as the most abundant protein source, distiller’s dried grains with solubles (DDGS) are the nitrogen-rich residues derived from the alcoholic beverage fermentation with maize, wheat, and sorghum [11]. DDGS receive a substantial income from the animal feed market, but have a low profit. The application of DDGS for production of value-added chemicals seems to be an incentive opportunity. Sugarcane vinasse in the bioethanol sector has a low content of proteins, and its economic value may be realized for an alternative application other than fertilizer [12]. As exploited for production of lignocellulosic biofuel, maize stover, wheat straw, and sorghum stove contain very few proteins. Interestingly, cassava leafs have a high protein content reaching around 40% w/w and provide a promising source of proteins. Figure 1 shows the crude protein content of selected biorefinery feedstock. The global production volume of soybean meal, rapeseed meal, and sunflower meal amounts to 200.8, 39.2, and 16 million metric tons (MMTs), respectively [13]. The production of maize DDGS and canola seed meal in US is around 23.1 (Renewable Fuels Association) and 1.07 MMTs [13], respectively.

### Alternative protein source

**Fig. 2:**
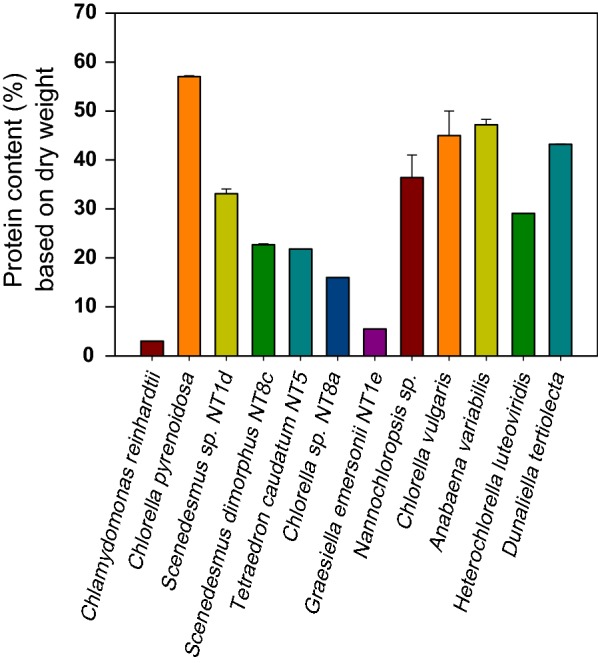
Protein content of selected microalgae. The various levels of proteins in selected microalgae are summarized

The volume of crops is largely limited by land availability. In contrast, microalgae which display a fast growth produce a high protein level and can be grown in open ponds. Microalgae approximately contribute to 40% of global photosynthesis by fixing CO_2_ [14]. There are more than 200,000 microalgae species on the planet, mostly including *Bacillariophyta* (diatoms), *Chlorophyta* (green algae), *Chrysophyta* (golden algae), and *Cyanophyta* (blue–green algae). The fractionated composition of algae generally comprises 35–50% proteins [15]. However, microalgae display high diversity in terms of ecological, metabolic, chemical, and biological characteristics. *Botryococcus braunii*, for instance, has hydrocarbons accounting for 75% of the total weight. *Diatom*, *Dunaliella salina*, and *Chlorella* sp. are rich in lipid ranging from 30 to 75% [16]. *Chlorophyta and Chlorophyceae* contain 60% proteins which can be finely processed into human nutrition source [17]. Chlorella strains cultivated in the photobioreactors outdoors enable production of 32 tons protein per year [18]. The composition of amino acids from microalgae is favorable for food, fishing, agriculture, and animal feed industry [17, 19]. As reported, the protein production of *Chlorella* reaches up to 400 g/g-biomass under the mixotrophic condition [20]. The protein content of selected microalgae is summarized in Fig. 2, including *Chlamydomonas reinhardtii* [21], *C. pyrenoidosa* [22], *Scenedesmus* sp. NT1d, *S. dimorphus* NT8c, *Tetrahedron caudatum* NT5, *Chlorella* sp. NT8a, *Graesiella emersonii* NT1e [23], *Nannochloropsis* sp. [24], *C. vulgaris* [20], *Anabaena variabilis* [25], *Heterochlorella luteoviridis*, and *D. tertiolecta* [26]. *C. vulgaris*, *D. bardawil*, *S. obliquus*, and *Spirulina platensis* have a well-balanced composition of amino acids [15]. Microalgal proteins are expected to contribute around 30% of the animal feed market in the future [27].

The protein content of fungi and bacteria is within a range of 30–70% and 50–80%, respectively [28]. Microbes have been genetically manipulated for mass production of amino acids and chemicals of specific interest [29], which is unaffected by climate changes. However, microbes contain a high level of nucleic acids and suffer a high risk of contamination with heavy metals and toxins [28]. Microbial proteins can provide an important and promising source if the contamination issue is well addressed.

## Protein recovery and hydrolysis

**Fig. 3:**
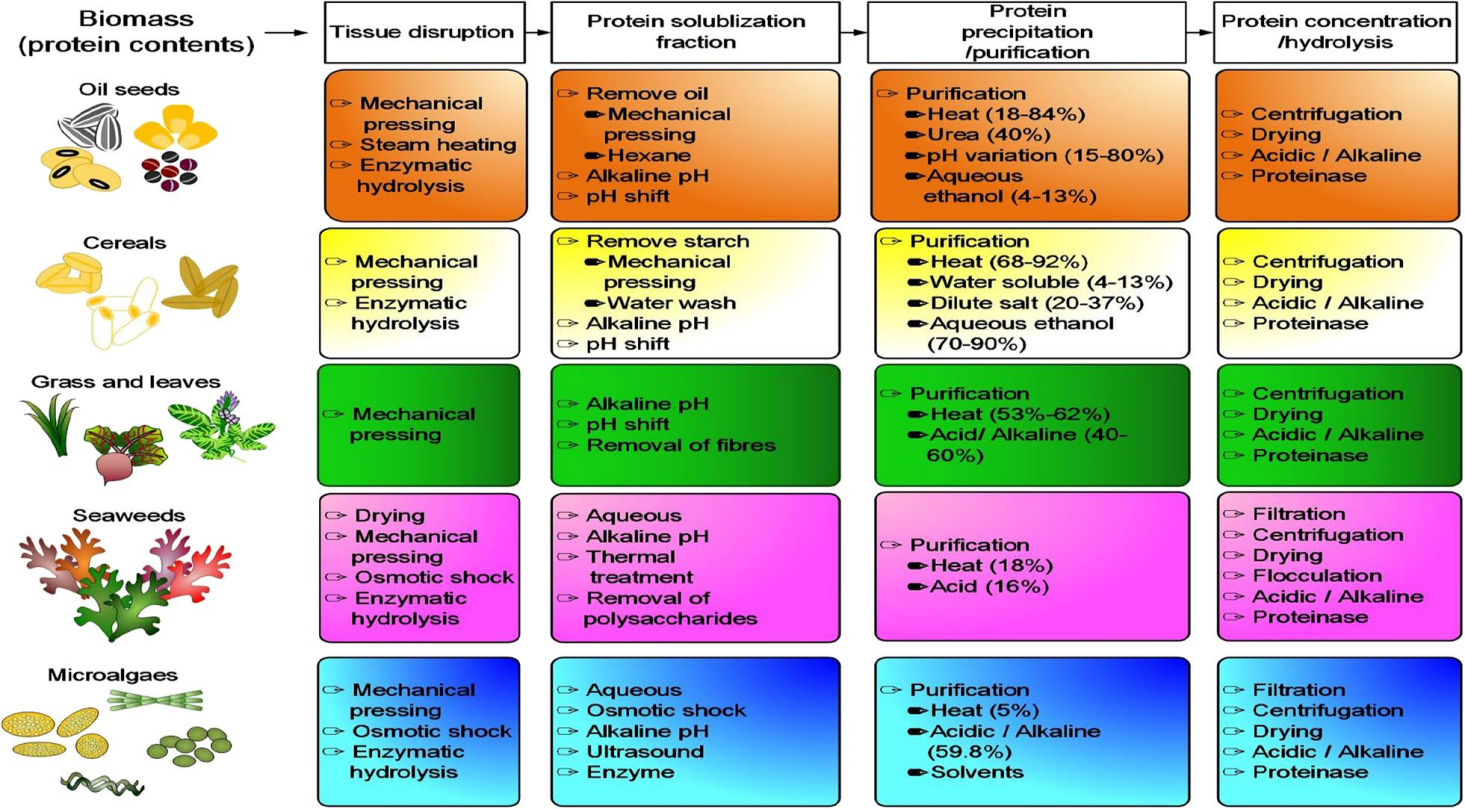
Flow chart of protein extraction and amino-acid recovery from various biomass sources. The treatment processes applied for selected feedstock are illustratively summarized. The numerical number in the parenthesis indicates the efficiency of the implemented methods

Recovery of protein waste from biomass feedstock is a necessary step prior to isolation of amino acids. Figure 3 outlines the treatment schemes applied for various feedstock including oil seeds [11, 30–33], cereals [11, 30, 31, 34, 35], grass and leaves [25, 30, 31, 36, 37], seaweeds [30, 31, 38], and microalgae [30–32, 39, 40]. The treatment process generally consists of (1) cell disruption and fractioning, (2) protein recovery, and (3) protein hydrolysis. It has been reported to purify oil seed proteins with the method of heat [41], urea [42], pH [43], and ethanol [44], and cereal proteins with heat [41], water soluble [45], salt and ethanol [46]. Grass and leaf proteins are recovered by heat [41] and acid/alkaline [38], seedweed proteins by heat [41] and acid [43], and microalgal proteins by heat [47] and acid/alkaline [38]. The purification efficiency of implemented methods varies with distinct feedstock (Fig. 3). Nevertheless, the alkali- or acid-based precipitation method is usually employed to separate proteins. An effective method was reported to precipitate proteins at pH 9 and 30 °C or pH 4.0–4.5 and 80–90 °C [48, 49]. One technology called ammonia fiber expansion (AFEX) illustrates efficient recovery of proteins from cellulosic biomass. The treatment process starts with the warm ammonia solution for extraction of proteins. Proteins are then obtained after drying [50]. Leaf protein processed by AFEX provides a viable method of alternative choice [51]. A convection method involves the use of a mechanical mill to disrupt leaf cells, followed by removal of the juice with a screw press. The juice is subject to heat to withdraw the coagulated proteins which are isolated after drying [52].

There are many methods developed to separate proteins from the waste stream in the biodiesel production process. One approach employs a multistep extraction method for recovery of *Jatropha* seed proteins [53]. *Jatropha* seed kernels and husks are first processed by either a milling machine or a screw press. The residue biomass after the machine pressing is extracted with solvents, followed by subjecting the crude extracts to three stages of cross-flow extraction. Proteins are finally isolated by precipitation at the acidic condition.

Disruption of cell walls presents to be the critical step in recovery of proteins from microalgae. This task is conventionally carried out by the chemical treatment using acid–alkaline solution (e.g., 0.4 M HCl and 0.4 M NaOH), or a two-phase system with polyethylene glycol (PEG) and potassium carbonate [54]. However, physical treatments usually favor the recovery of microalgal proteins at a large scale. Many methods have been developed, including high-pressure homogenization, liquid nitrogen grinding, ultrasonic crushing, osmotic cracking pulsed electric field, and microwave-assisted extraction [55, 56]. Moreover, other cell compositions associated with proteins are removed by precipitation or fractionation to ensure a good quality of recovered proteins [57]. Nevertheless, the application of microalgal proteins still remains to explore.

The hydrolysis of proteins is commonly carried out with a prolonged treatment of acids or alkalis. However, not all amino acids remain intact during the treatment process [58, 59]. A milder way lies in the use of proteases. Alcalase is an alkaline protease and has been exploited for hydrolysis of proteins in poultry, shrimp waste, and wheat gluten [60–62]. In addition, proteins extracted from wheat DDGS were treated with protease including Protex 14L, Protex 6L, and Protex 51P [63]. The production of amino acids from sorghum was proven feasible by the amino-peptidase and the neutral proteinase from Novozymes [64]. A patent discloses the employment of peptidases to release free amino acids from protein sources [65]. Lactic acid bacteria possess a variety of peptidases [66], and have a potential application for production of chemicals (see “Discussion and future perspectives”). Enzymatic reactions require a controlled condition to optimally proceed, which may be fulfilled by the pH-stat enzymatic hydrolysis [67].

## Catabolism of proteinogenic amino acids

**Fig. 4:**
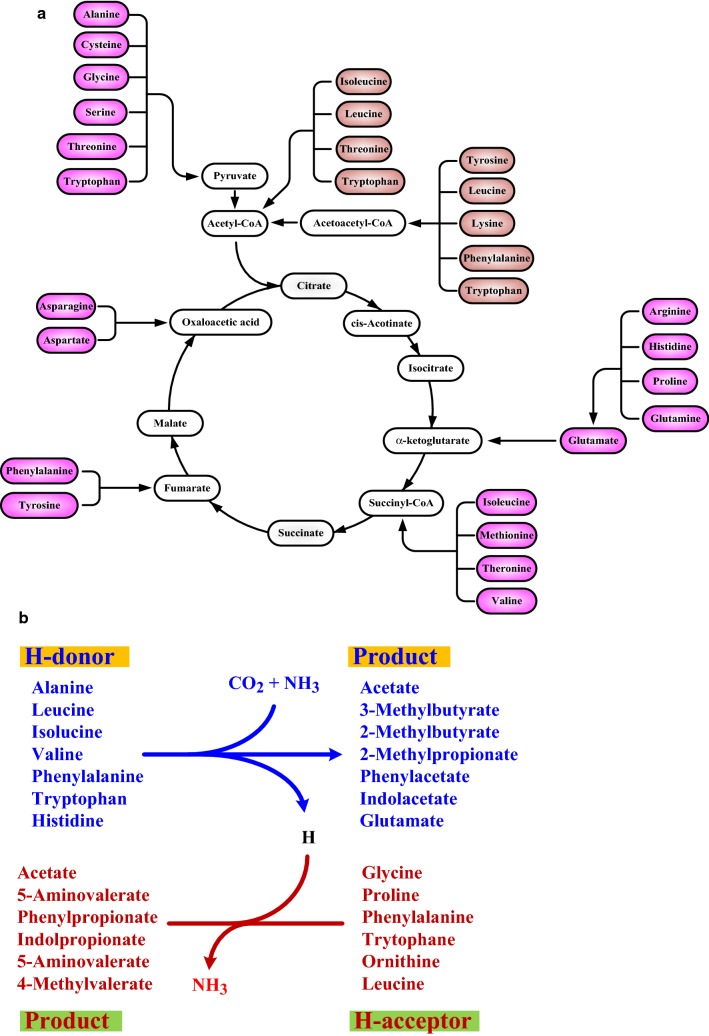
Catabolism of proteinogenic amino acids. **a** Catabolic routes of amino acids linking to the central metabolism. **b** Involvement of H donors and H acceptors in Stickland reactions. In the oxidation reaction, alanine, leucine, isoleucine, valine, phenylalanine, tryptophan, and histidine function as H donors to produce acetate, 3-methylbutyrate, 2-methylbutyrate, 2-methylpropionate, phenylacetate, indolacetate, and glutamate, respectively. In the reduction reaction, glycine, proline, phenylalanine, tryptophan, ornithine, and leucine function as H acceptors to produce acetate, 5-aminovalerate, phenylpropionate, indolpropionate, 5-aminovalerate, and 4-methylvalerate, respectively

The synthesis of 20 proteinogenic amino acids requires six precursor metabolites in the central metabolism, involving glycolysis, the tricarboxylic acid (TCA) cycle, and the pentose phosphate (PP) pathway. Inorganic ammonia is assimilated by reductive amination of α-oxoglutarate with l-glutamate dehydrogenase (GDH) in *Escherichia coli*. The amination of glutamate mediated by l-glutamine synthetase (GS) leads to glutamine. Moreover, ammonia in low concentration is assimilated to glutamate by the combined reaction of GS and glutamate synthase (or glutamine: α-oxoglutarate aminotransferase). Glutamate and glutamine serve as ammonia donor for reductive synthesis of more than 10 amino acids. The catabolism of proteinogenic amino acids differs from their anabolic pathways due to the thermodynamic constraint and subtle molecular regulations. In general, the catabolic route for glucogenic amino acids leads to pyruvate and metabolite nodes in the TCA cycle and for ketogenic amino acids ends with production of acetyl-CoA and acetoacetyl-CoA (Fig. 4a). The catabolism of amino acids varies in living cells. This review is mainly focused on the catabolic pathways in *E. coli* and selected microbes.

### Amino-acid catabolism with single or two steps

In *E. coli* and *Saccharomyces cerevisiae*, glycine is oxidatively degraded to CO_2_, ammonia, and a methylene group by the glycine cleavage complex (namely, glycine decarboxylase) [1, 68]. The methylene group enters one-carbon metabolism mediated by tetrahydrofolic acid (THF). Consequently, the formation of methylene-THF drives the serine synthesis by serine hydroxymethyl transferase in *E. coli*. Alternatively, *Clostridium sticklandii* employs a glycine degradation route consisting of glycine decarboxylase and glycine reductase to produce acetate [69].

Serine is deaminated to pyruvate by serine deaminase in *E. coli* [70] or serine dehydratase in *S. cerevisiae* [71]. *E. coli* synthesizes two serine deaminases (encoded by *sdaA* and *sdaB*), but is unable to utilize serine as sole carbon source.

Cysteine is a sulfur-containing compound and degraded to pyruvate and H_2_S in *E. coli*. There involves cysteine desulfhydrase (encoded by *cysK* and *cysM*) in this catabolic reaction [72]. In addition, tryptophanase (encoded by *tnaA*) displays a catalytic function of cysteine desulfhydrase and plays a key role in cysteine utilization. Its synthesis is induced in the presence of tryptophan [73].

*E. coli* utilizes alanine in a unique way. l-Alanine is first converted to d-alanine by alanine racemase (encoded by *dadX* and *alr*). A subsequent reaction catalyzed by d-amino-acid dehydrogenase (encoded by *dadA*) of broad substrate specificity leads to deamination of d-alanine to pyruvate [74]. This catabolic pathway enables *E. coli* to utilize both l-alanine and d-alanine. Alternatively, *Bacillus subtilis* obtains the energy for sporulation by conversion of l-alanine to pyruvate with alanine dehydrogenase [75].

The synthetic pathway of aspartate is reversible and mostly adopted for its utilization in living cells. By the aspartate aminotransferase (encoded by *aspC*)-mediated transamination reaction, aspartate is converted to oxaloacetate and α-ketoglutarate receives the amino group to give glutamate. Aspartate is also deaminated to fumarate as catalyzed by aspartase in *E. coli* and lactic acid bacteria [76, 77].

The degradation of asparagine is initiated by its conversion to aspartate as catalyzed by asparaginase. Further catabolism of aspartate proceeds with aspartase. *E. coli* enables synthesis of two asparaginases encoded by *ansA* and *ans*B [78].

The utilization of glutamate is limited by its inefficient transport in *E. coli*. The AspC-catalyzed transamination reaction mediates the conversion of glutamate to α-ketoglutarate and aspartate. Aspartate is deaminated to fumarate catalyzed by aspartase [79]. In *S. cerevisiae*, the NAD-dependent glutamate dehydrogenase serves as the main pathway for degradation of glutamate to α-ketoglutarate and ammonia [80].

*E. coli* exhibits slow growth on glutamine. Glutamine is utilized by conversion of two glutamate based on glutamate synthase, followed by glutamate catabolism [81].

Hydrolysis of proline to glutamate proceeds in two steps. *E. coli* synthesizes proline dehydrogenase (encoded by *putA*) with a dual function which is responsible for this catabolic pathway [82].

*E. coli* has two aerobic degradation routes of threonine [83]. The major route consists of threonine dehydrogenase (encoded by *tdh* or *yiaY*) and 2-amino-3-ketobutyrate CoA ligase (encoded by *kbl*) and enables conversion of threonine to glycine and acetyl-CoA. Another route comprises low-specificity l-threonine aldolase (encoded by *ybjU*) which generates glycine and acetaldehyde from threonine.

### Amino-acid catabolism with multiple steps

The catabolic pathway of histidine consists of four reactions steps, which leads to the formation of glutamate and formamide. This pathway exists in many microbes (such as *B. subtilis*) but not found in *E. coli* [84].

Microbes utilize arginine through various pathways [85]. The catabolic reaction starts with arginine succinyltransferase or arginine decarboxylase in *E. coli*, arginine oxidase in *P. putida*, arginine deiminase in lactic acid bacteria, arginase in *B. subtilis*, and arginine:pyruvate transaminase in *P. aeruginosa*. The arginine succinyltransferase (AST) pathway is the major catabolic pathway of arginine in *E. coli*. The α-amino group of arginine is first succinylated, followed by deamination of two amino groups and transamination of the third amino group on the side chain. The succinyl group is detached from the α-amino group of arginine through hydrolysis, finally giving glutamate and succinate. The arginine deiminase (ADI) pathway found in *C. sticklandii* [69] and lactic acid bacteria [66] produces intermediate metabolites including citrulline, ornithine, and carbamoyl phosphate which appear in the synthetic pathway of arginine. Carbamoyl phosphate is further cleaved into CO_2_ and ammonia with generation of ATP. This substrate-level phosphorylation in arginine catabolism is coupled with the bacterial growth [66].

Leucine, isoleucine, and valine are branch-chain amino acids (BCAAs) which produce volatile compounds involving acids, aldehydes, alcohols, and esters. Their degradation starts with the transamination reaction using α-oxoglutarate as the amino acceptor to produce α-oxoisocaproate, α-oxomethylvalerate, and α-oxoisovalerate, respectively. Aminotransferases such as AraT (EC 2.6.1.1) and BcaT (EC 2.6.1.42) in lactic acid bacteria and BAT1 in *S. cerevisiae* are responsible for this transamination reaction [86, 87]. The degradation pathways for conversion of these α-oxoacids to aldehydes, carboxylic acids, and hydroxyacids are common in most microbes. Alternatively, the common pathway for conversion of leucine, isoleucine, and valine to 3-methylcrotonyl-CoA, (*E*)-2-methylcrotonoyl-CoA, and methylacrylyl-CoA proceeds with BCAAs aminotransferase, branched-chain α-ketoacid dehydrogenase, and 2-methylacyl-CoA dehydrogenase, respectively. The subsequent cleavage of these acyl-CoA derivatives goes through distinct routes, consequently leading to propionyl-CoA, acetyl-CoA, or acetoacetate.

Tryptophan can be degraded in various ways. *E. coli* synthesizes tryptophanase which catalyzes the conversion of tryptophan to indole, pyruvate, and ammonia [88]. The catabolic pathway involving homogentisate as an intermediate is employed by animals, fungi, and some bacteria for tyrosine degradation. Further hydrolysis of homogentisate produces fumarate and acetoacetate. The degradation of phenylalanine can proceed with the same pathway after its hydroxylation to tyrosine by phenylalanine hydroxylase [89].

The degradation pathway of lysine diversifies. *P. putida* catabolizes lysine to glutarate via the δ-aminovalerate pathway [90]. Glutarate is activated to glutaryl-CoA which is cleaved to CO_2_ and acetyl-CoA in several steps [91].

Note that *S. cerevisiae* has evolved a generalized pathway for utilization of amino acids discovered by Ehrlich [92]. The Ehrlich pathway starts with the transamination reaction by conversion amino acids to their respective α-keto acids. Subsequent decarboxylation of α-keto acids produces aldehydes, known as fusel aldehydes. Fusel aldehydes are finally reduced to fusel alcohols or oxidized to fusel acids, which serve as flavor compounds or precursors of flavor compounds. The Stickland reaction prevails in clostridial species that ferment the amino-acid mixture [93]. In this reaction, some amino acids preferably function as H donors, while others H acceptors (Fig. 4b). It is carried out by coupling the oxidation reaction of one amino acid with the reduction reaction of another. For instance, the oxidation of glycine pairing with the reduction of glycine produces acetate and ammonia. This reaction is featured with conservation of ATP via the substrate-level phosphorylation.

## Bio-based production of fuels and chemicals from amino acids

### Biotransformation approach

Protein hydrolysates contain a mixture of 20 amino acids. Isolation of amino acids is required for performing biotransformation. Many methods for separation of a single amino acid from a mixture have been developed, and each has its own advantage and disadvantage. By the electrodialysis method, amino acids are separated into the acidic, basic, and neutral groups [94]. The potential problem is the interaction of some amino acids with the ion-exchange membrane. A recent study has reported the use of ethanol to fractionally precipitate amino acids [57]. Groups of amino acids are separated from a mixture, which needs extra work for the complete separation of amino acids. The implementation of chromatography appears useful for isolation of individual amino acids [95]. However, these mentioned methods are generally impractical due to a high cost associated with the scale-up operation and waste management.

A variety of amino group-containing compounds are idea candidates for production from amino acids with a simple reaction scheme. Learning from the amino-acid catabolism of microbes, the deamination, decarboxylation, and hydrolysis reactions provides a basis for the production scheme (Table 1). A good example illustrates the arginase-catalyzed conversion of arginine to ornithine [96]. The starting material for Nylon-4,6 can be obtained by further decarboxylation of ornithine to 1,4-diaminobutane using ornithine decarboxylase [97]. This two-step reaction is limited by ornithine decarboxylase [total turnover number (TTN) of ~ 10^5^] due to its lower operational stability than arginase (TTN of ~ 10^9^). The phenylalanine ammonia lyase (PAL)-mediated reaction produces cinnamic acid from phenylalanine [98]. The importance of cinnamic acid is acknowledged as the precursor for the synthesis of styrene (> 1.7 × 10^7^ tons/year). PAL prevails in yeast and is subject to oxidation. The reaction scheme is usually conducted with suspended whole cells under the anaerobic and static condition. A similar idea can also be applied for production of β-alanine by decarboxylation of aspartate with aspartate α-decarboxylase [99]. β-Alanine has a potential application for the synthesis of acrylonitrile and acrylamide (> 0.5 × 10^6^ tons/year). This enzymatic reaction generates CO_2_ which causes a pH shift and the malfunction of the fixed-bed reactor. The oxidation and decarboxylation of lysine lead to 5-aminovaleric acid and 5-diaminopentane, respectively. The former reaction proceeds with lysine oxidase, while the latter with lysine decarboxylase [100–102]. The potential application of 5-aminovaleric acid and 5-diaminopentane is their use for production of nylon and polyamide. However, the enzymatic reaction is less efficient and conducted for several days to achieve a conversion yield of 95%. Glutamic acid is a non-essential amino acid and the most abundant amino acid found in most of feedstock proteins. Through the decarboxylation reaction, glutamate is converted to γ-aminobutyric acid by glutamate decarboxylase (GAD) [103]. The synthetic routes starting from γ-aminobutyric acid to *N*-methylpyrrolidone (NMP) and *N*-vinylpyrrolidone (NVP) are considered environmentally favorable. NMP and NVP are useful intermediates for the synthesis of many bulk chemicals. The GAD-mediated reaction is relatively efficient with a complete conversion of glutamic acid within 3 h, which gives a production rate of 34.3 g/L/h. The deamination of glutamate by glutamate deaminase gives α-ketoglutaric acid [104, 105]. Interestingly, α-ketoglutaric acid can be polymerized into poly(triol α-ketoglutarate), a biodegradable material. However, this enzyme displays instability to lose 75% of the activity after the four times reuse of the immobilized cells.

**Table 1:** Summary of selected biotransformation of amino acids into chemicals

Amino acid	Product	Enzymatic reaction	Enzyme
Arginine	Ornithine		Arginase
Aspartic acid	β-Alanine		Aspartate α-decarboxylase
Glutamic acid	α-Ketoglutaric acid		Glutamate deaminase
Glutamic acid	*N*-Methyl- and *N*-vinyl-pyrrolidone		Glutamate decarboxylase and NADH oxidase
Lysine	5-Aminovaleric acid		Lysine oxidase
	5-Diaminopentane		Lysine decarboxylase
Phenylalanine	Cinnamic acid		Phenylalanine ammonia lyase
Alcohol and alanine	Primary amines		Alcohol dehydrogenase (ADH-hT), ω-transaminase (ωTA) and l-alanine dehydrogenase (AlaDH)
l-Amino acids	Enantio-compounds		l-Amino acid oxidases (l-AAD), isocaproate reductase (Hic) and formate dehydrogenase (FDH)

Primary amines are important for the synthesis of azo dyes, antioxidants, or rubber products. They can be obtained from primary alcohols using one-pot enzymes including thermostable alcohol dehydrogenase (ADH-hT), ω-transaminase (ωTA), and l-alanine dehydrogenase (AlaDH) [106]. ADH-hT participates in the oxidation reaction, while ωTA catalyzes the amination reaction. The oxidation and amination reactions are continued to occur by cycling l-alanine/pyruvate and NAD^+^/NADH with the aid of AlaDH. The conversion reaction can be driven to completion (up to 99%) using ammonia as an amino-donor in the presence of 1,2-dimethoxyethane at room temperature. An alternative approach to obtain amine has been illustrated with galactose oxidase from *Fusarium* sp., and a ω-TA from *B. megaterium*, *P. putida*, *Paracoccus denitrificans*, and *Vibrio fluvialis* [107]. Like AlaDH, either formate dehydrogenase or glucose dehydrogenase was employed to complete the l-alanine/pyruvate and NAD^+^/NADH cycle [108]. The continued operation of these reactions is driven by regeneration of NAD^+^ which is added as a cofactor.

l- or d-Hydroxy acids mostly used for enantio-drugs can be synthesized by a designed cascade reaction, namely one-pot simultaneous multi-enzyme system [109]. The (*R*)- and (*S*)-enantiomers of amino acids are converted to enantiopure form (> 99% ee) by l-amino-acid oxidases (l-AAD) [110]. A subsequent reaction proceeds as an asymmetric reduction which consists of the coupled reaction of isocaproate reductase (Hic) and formate dehydrogenase (FDH) [111]. The implementation of l- or d-Hic enables selective production of the hydroxy acid enantiomers. The regeneration of NADH cofactor is carried out by FDH, which is widely applied in industry.

### Pathway engineering approach

**Fig. 5:**
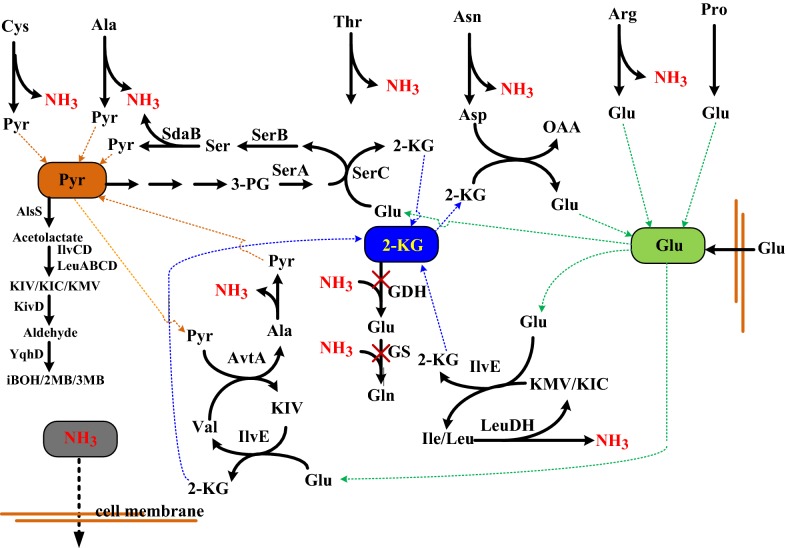
Rewiring of metabolic pathways in *E. coli* for conversion amino acids to biofuels and ammonia. The rational design of catabolic pathways for amino acids leads to the accumulation of intracellular metabolites involving pyruvate, α-oxoglutarate, glutamate, and ammonia. The deleted genes are marked with “X”. *OAA* oxaloacetate, *Pyr* pyruvate, *2-KG* α-oxoglutarate, *KIV* 2-ketoisovalerate, *KIC* 2-ketoisocaproate, *KMV* 3-ketomethylvalerate, *iBOH* isobutanol, *2-MB* 3-methyl-1-butanol, *3MB* 3-methyl-1-butanol

Naturally occurring microbes exhibit a different degree of amino-acid utilization as the carbon and nitrogen source. Nevertheless, the production of chemicals by microbes using protein waste can be implemented without the need for isolation of amino acids. This idea has been well illustrated by pathway engineering of *E. coli* to rewire and optimize its amino-acid catabolism [112]. *E. coli* was first evolved to utilize 13 individual amino acids after several rounds of chemical mutagenesis. To produce isobutanol, the evolved strain was endowed with the synthetic pathway. The microbial consumption of proteins may be restricted upon induction of quorum sensing in cells grown on the protein-rich medium [113]. The regulatory circuit of amino-acid catabolism was then disabled by deletion of the quorum-sensing genes *luxS* or *lsrA*, consequently increasing the isobutanol production. Furthermore, a driving force to drain more amino acids was created by blockage of the nitrogen assimilation pathways involving GDH and GS (Fig. 5). This approach led to the accumulation of glutamate and BCAAs. Three transamination–deamination cycles were generated for utilization of BCAAs by overexpression of *leuDH* from *Thermoactinomyces intermedium* and endogenous genes involving *ilvE*, *avtA*, and *sdaB*. The first cycle operates using LeuDH to deaminate isoleucine and leucine to 2-keto methylvalerate and 2-keto isocaproate, respectively. The second cycle driven by AvtA converts valine to 2-keto isovalerate, and pyruvate accepts the amino acceptor of valine. Through the IlvE-catalyzed transamination reaction, these three α-keto acids receive the amino group from glutamate to form isoleucine, leucine, and valine, respectively. The third cycle proceeds by SdaB-mediated deamination of serine to pyruvate. Re-synthesis of serine occurs by gluconeogenic conversion of pyruvate to 3-phosphoglycerate which serves as the precursor for serine synthesis via the native synthetic pathway consisting of *serA*, *serB*, and *serC*. These combined strategies finally resulted in the production of biofuels (isobutanol, 2-methyl-1-butanol, and 3-methyl-1-butanol) accounting for 56% the theoretical yield. Another merit of this approach is manifested by removal of the ammonia from amino acids, and the recycled nitrogen may provide the need of fertilizer for crops. This reduces the dependence of chemical fertilizer stemming from the environmentally unfavorable Haber–Bosch process, which eventually ameliorates the climate change [114]. Recently, the same idea has been applied for production of biofuels from DDGS by a bacterial consortium which consists of two strains designed for selective utilization of carbohydrates and amino acids, respectively [115]. The strategy by deamination of amino acids has also been exploited for the production of ammonia [116].

Although feasible, the amino acid-based production scheme of biofuels by genetically modified *E. coli* is afflicted with the need for protein hydrolysate. The implementation of a native protease-secreting microbe such as *B. subtilis* seems to provide a solution to this issue by integration of protein hydrolysis and amino-acid fermentation in one step. The strategy exploited for pathway engineering of *B. subtilis* was carried out in several steps [117]. In essence, three transamination–deamination pathways for cycling BCAAs were established by recruitment of *T. intermedium leuDH*. Two cycle pathways generally resemble those in *E. coli*. One involves YbgE (like *E. coli* IlvE) and LeuDH to cycle isoleucine, leucine, and valine. Valine is cycled in another route by coupling YbgE with AlaT (like *E. coli* AvtA). The third cycle proceeds with deamination of glutamate by endogenous RocG and re-synthesis of glutamate by YbgE-mediated transamination. Moreover, the regulatory circuit involved in the related pathways of BCAAs was decoupled by removal of the global transcriptional regulator CodY. Inactivation of BkdB to nullify the degradation pathway of BCAAs led to accumulation of 2-keto acid pools. Finally, the engineered *B. subtilis* enabled production of biofuels with 18.9% of the theoretical yield after recruitment of the Ehrlich pathway comprising *Lactococcus lactis kivD* and *E. coli yqhD*.

## Discussion and future perspectives

The world is now entering a new era of the bio-based economy that is marked with sustainability and ecological soundness. To support the growth of this economy system, the biorefinery platforms need to be continuously advanced. The historical production of bio-based fuels and products has been mainly carried out with sugar feedstock. However, the cost of bio-based products generally exceeds that of petrochemical counterparts. A considerable volume of proteins will be generated in the waste streams associated with the sugar-based production process. Therefore, the economical viability of the biorefinery platform can be realized by incorporation of the protein-based production scheme into the existing production scheme based on sugars. This scenario is well exemplified by a recent study reporting the microbial production of C4 and C5 fusel alcohols from DDGS [115]. An *E. coli* strain which metabolizes glucose and xylose was equipped with the synthetic pathway of fusel alcohols by overexpressing Als, IlvCD, KivD, and YqhD (refer to Fig. 5). In addition to the synthetic pathway of fusel alcohols, the catabolic pathways of amino acids were rewired in another *E. coli* strain for high utilization of amino acids according to the reported approach [112]. Sugars and amino acids are released in DDGS by pretreatment with dilute sulfuric acid and Pronase. Consequently, co-culturing of the two strains on DDGS hydrolysate enables production of 10.3 g/L fusel alcohols. The production of putrescine by recombinant *C. glutamicum* presents another example. By expressing ornithine decarboxylase, this strain was grown on glucose, while supplemented with l-arginine as a precursor [118]. An earlier study proposed a process to fractionate sugarcane leaves and tops for simultaneous production of electricity, single cell proteins, and leaf proteins [119]. It would be appealing to integrate this process with a production scheme of chemicals based on leaf proteins. Nevertheless, the development of the protein-based biorefinery is still immature and fallen far behind the biomass-based platform.

The biotransformation method provides a simple way for production of bulk chemicals from amino acids. However, isolation of a single amino acid from protein hydrolysates is required and remains technically difficult. One potential solution is provided by the microbial production of cyanophycin [120, 121]. Cyanophycin consists of a poly-l-aspartic acid backbone with l-arginine side chain. The employment of recombinant strains grown on sugars enables mass production of insoluble cyanophycin by direct utilization of aspartic acid and arginine present in the medium. Either aspartic acid or arginine recovered from the hydrolysis treatment of isolated cyanophycin is readily applicable for the biotransformation production. Apparently, this approach provides a useful route for selective separation of aspartic acid and arginine from others in protein waste. The industrial production of l-glutamic acid by microbial fermentation has been practiced over 50 years ago since its birth [122]. Until now, almost all proteinogenic amino acids can be produced with rationally designed microbes on industrial scale [123]. Based on this well-established fermentation scheme, the amino-acid producer strain may be modified for utilization of surplus or non-essential amino acids. Recovery of the produced amino acid is made possible with crystallization [124]. In addition, the stability of enzymes appears to be another challenge that limits the development of biotransformation. The method using directed evolution or/and immobilization would be useful to address this issue [125, 126].

Naturally existing microbes are evolved to utilize part of amino acids and produce fermentation products such as hydrogen and n-butanoate/acetate [127, 128]. As described in “Catabolism of proteinogenic amino acids” section, it seems possible to engineer a microbe for utilization of all amino acids by integration of the catabolic pathways from a variety of strains. One of various catabolic pathways for a specific amino acid can be chosen for design according to the engineering purpose. The catabolic routes of amino acids interconnect the central metabolism at distinct nodes (Fig. 4a). To direct the carbon flux to the desired pathway of a specific product, the task necessitates a rational design of pathways and is highly delicate and complicated to fulfill. A consolidated platform for production of chemical is appealing without the need of processing proteins into amino acids. An illustrative example employs *Lactococcus lactis* (naturally secreting proteases) for nisin production based on defatted soybean meal [129]. Amino acids from intracellular hydrolysis of small peptides were utilized to synthesize nisin. A synthetic consortium may be designed by co-culturing of one protease-secreting strain and another strain specialized in utilization of amino acids. This approach will simplify the genetic manipulation and ameliorate the metabolic stress incurred by overexpression of many enzymes.

**Fig. 6:**
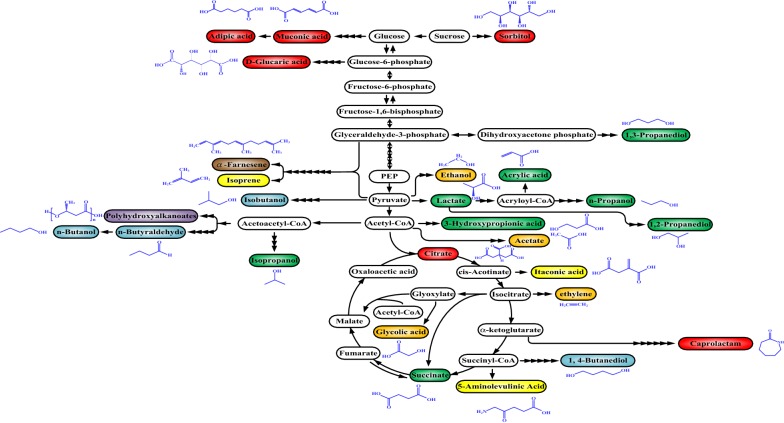
Biomass-derived building blocks of industrial interest. The schematic drawing illustrates the synthetic pathways leading to building blocks of industrial interest

Figure 6 shows the biomass-derived building blocks of chemicals that receive industrial interest. All of them are either in development or in pipeline. The list includes C-2 products (e.g., ethanol [130], ethylene [131], and glycolic acid [132]), C-3 products (e.g., lactate [133], 1,3-propanediol [134], isopropanol [135], 3-hydroxy propanoic acid [136], 1,2 propanediol [137], acrylic acid [138], and n-propanol [139]), C-4 products (e.g., n-butanol [140], isobutanol [141], n-butyraldehyde [142], 1,4-butanediol [143], and succinate [144]), and products with a high carbon number (*n* > 6) (e.g., muconic acid [145], itaconic acid [146], isoprene [147], 5-aminolevulinic acid [148], adipic acid [149], glucaric acid [150], caprolactam [151], α-farnesene [152], and polyhydroxyalkanoates [153]). In general, the synthetic routes of these terminal products starting with sugars can be transformed into those based on amino acids. Through the catabolic pathways, the carbon-skeleton flux of either glucogenic amino acids or ketogenic amino acids is re-directed into the bifurcated pathways of interest. In addition, the introduction of the CO_2_-fixation pathways [154] into producer strains may improve the production yield due to the involvement of decarboxylation of amino acids.

In conclusion, the production of bio-based chemicals and polymers is fallen far short of the petrochemical production volume. This demands that researchers across interdisciplinary areas work together to accelerate the pace of R&D and come up with novel biorefinery platforms. The bio-based economy is beginning to reshape our life.

## Abbreviations

ADH-hT: alcohol dehydrogenase
ADI: arginine deiminase
AFEX: ammonia fiber expansion
AlaDH: l-alanine dehydrogenase
AST: arginine succinyltransferase
BCAAs: branch-chain amino acids
DDGS: distiller’s dried grains with soluble
FDH: formate dehydrogenase
GDH: l-glutamate dehydrogenase
GS: l-glutamine synthetase
l-AAD: l-amino-acid oxidases
l-Hic: isocaproate reductase
NMP: *N*-methylpyrrolidone
NVP: *N*-vinylpyrrolidone
PP: pentose phosphate
TCA: tricarboxylic acid
THF: tetrahydrofolic acid
ωTA: ω-transaminase
